# Anthropogenic interventions on land neutrality in a critically vulnerable estuarine island ecosystem: a case of Munro Island (India)

**DOI:** 10.1038/s41598-023-28695-w

**Published:** 2023-01-26

**Authors:** M. K. Rafeeque, T. R. Anoop, M. K. Sreeraj, R. Prasad, L. Sheela Nair, A. Krishnakumar

**Affiliations:** 1 National Centre for Earth Science Studies, Thiruvananthapuram, India; 2 University of Kerala, Thiruvananthapuram, India

**Keywords:** Geomorphology, Sustainability, Environmental impact, Restoration ecology

## Abstract

All landscapes, including estuarine islands, normally try to restore their geomorphic isostasy in all anthropogenic interventions on land dynamics. Munroe Island has been experiencing drastic environmental degradation, such as land subsidence, severe tidal/monsoon flooding, subsidence of build-ups and a drastic decay in agricultural productivity. This paper examines the role of anthropo-geomorphic interventions causing for the land degradation in Munroe Island through a multidisciplinary approach. Multidated, multiresolution satellite products and published maps, spanning a period of about six decades from 1960 to 2021, were used to understand the different geomorphic and geographical processes in the study area. Evaluation of the temporal bathymetric datasets, salinity measurements of the river and estuary, borehole data logs of the area and electrical resistivity surveys of the island were analyzed to find out the causative factors for the disturbances in the land neutrality, along with the tidal hydrodynamic changes in the region. The study shows about 14% of the total land area was vanished during the study period, and more than 25% of the area is under stress, leading to further land degradation. More than 500 households are forced to vacate their residence due to land subsidence/flooding. Lack of required freshwater and sediment supply from the Kallada river after the construction of the *Thenmala* reservoir in the Kallada river as well as the uncontrolled sand mining prevailed are the key factors for the environmental degradation of Munroe Island. The paper describes the role and colinkages of human-induced hydrogeomorphic interventions on a geomorphic system, in charge of the environmental degradation and land subsidence crisis of an estuarine island ecosystem and discusses the concerns related to the management strategies of such region.

## Introduction

Many estuarine islands and low-lying coasts are critically endangered by environmental degradation due to human-induced climatic and geomorphic disturbance. A recent study by Gerardo et al.^1^*.* revealed China, Indonesia and Japan are the most widely affected nations by land vulnerability issues and about 200 locations over 34 countries of the globe are threatened by land degradation due to groundwater depletion and associated hydro-morpho dynamics. Hydro-morpho dynamics and groundwater fluctuations affect landscape modification through erosion-accretion processes. The estuarine environments are regulated by the geomorphic actions like continuous land–sea interaction, intensive sedimentation, sea level fluctuations, saline water intrusion and coastline erosion, as well as various human activities such as coastal reclamation, oil extraction, urbanization, and agricultural activities^2–8^*.* An Island is not only the storage pool of important ecological functions and the living carrier of human beings, but also the platform of earth conservation and exploitation^9,10^. The areas of lowlands and floodplains of river valleys and coastal wetlands, especially estuarine regions, are more threatened by land degradation problems, as the geomorphological agents in such areas are more dynamic. A classic example is Lohachara Island's disappearance^11^, which was part of the delta in the Sundarbans National Park, West Bengal, India. It was an inhabited island where more than 6000 people lived until it was permanently flooded in the 1980s. Few more islands of the Sundarban delta, such as *South Talpatti,* extending about 210 km^2^ and *Ghoramara*, have disappeared due to sea-level rise and associated morpho-dynamic activities^12^ in recent years. Satellite images and sea patrols further confirmed the disappearance of these islands. Similar scenarios are also observed in the densely populated coastal plains of Florida state from Miami to Jacksonville (vital tourism destination of the United States) affected by frequent flooding and tide-induced gully erosion. On the other hand, the Miami coast of North America is frequently affected by sunshine flooding, due to sea level rise and urbanization^13^.

**Figure 1:**
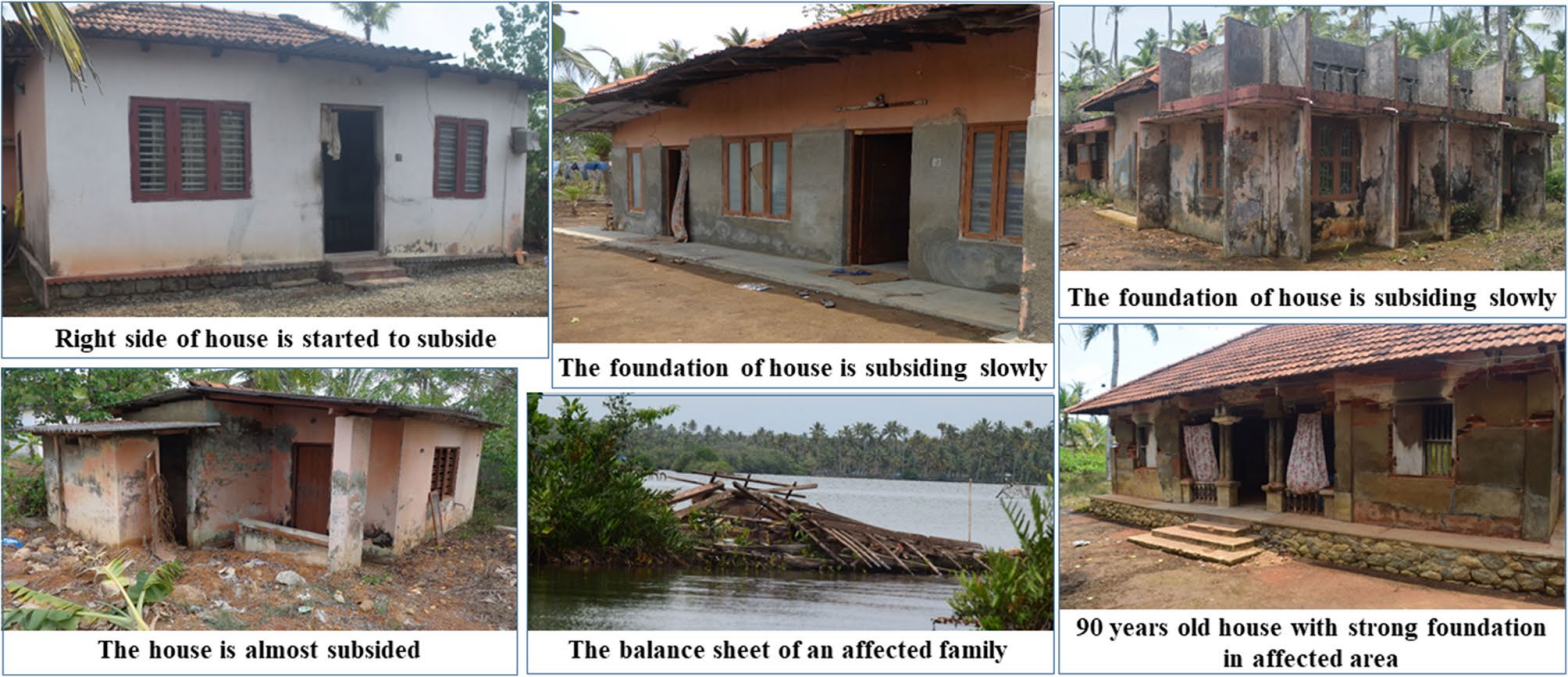
Environmental threat due to land subsidence in Munroe Island. Photographs taken by Rafeeque MK.

Rising sea level due to climate change threatens the wetland systems and the nearby land areas are causing the drastic geo-morphological changes. There are many driving factors for the land degradation of wetland regions, including the impact of climate change, sea level rise, and neotectonic activities. The direct causes of land degradation/land subsidence are due to unscientific removal/ deforming of Earth's surface materials, natural or human-induced geomorphological imbalances caused by changes in denudational processes, extreme weather disturbances, over pumping of groundwater, leakages of oil and gas from underground reservoirs/organic soils, underground erosion and the collapse of underground aquifers and mines. Tidal and saline water intrusion causes soil distortion and coastal vulnerability due to sea level rise^14^. The drastic changes in the hydrogeomorphic characteristics of coastal wetlands disturb the sustainability of coastal biodiversity and affect the isostatic existence of coastal wetlands. The augmentation of environmental distortion due to extreme weather phenomena such as cyclones, hurricanes, flash floods and anthropogenic influence in redefining denudational activities such as choking or diverting natural flows, the construction of bunds/ dams and mining, etc., will be setting up background conditions for land degradation considerably. There are many cases repeated from various places. Overexploitation of groundwater caused significant damage to buildings and infrastructure in the Mexico City^15^; islands of Tuvalu or Kiribati and their embattled archipelagos have been affected by climate change^16^, and sunshine floods or Miami rise have resulted from sea level rise^13^. Sahu and Sikdar^17^, Suganthi et al.^18^ and Harms^11^ studied the South Bengal basin's land subsidence issues. They concluded that along with the crucial factor of groundwater withdrawal, geological and anthropological factors were also played a major role for the subsidence. Munroe Island, a group of brackish islands and islets located in conjunction of the Kallada river and Ashtamudi backwater of Kerala along the SW coast of India, has been experiencing a drastic shrinkage of its area over the years (Fig. 1). Ashtamudi backwaters and associated islands are designated as protected wetlands for the conservation and sustainable use of wetlands as per the Ramsar convention in 2002^19^. Soil erosion due to tidal/monsoon flooding and the subsidence of constructed structures are the severe environmental issues occurring in the Munroe Island and the existence of these islands is at stake.

Various studies were conducted to understand the geo-environmental issues faced by the inhabitants of the Munroe Island group and some of them concluded as the Tsunami of 2004 is the main cause of Island’s land degradation while others derived the causes of issues as the effect of sea-level rise and anthropogenic activities. *Madhusoodhanan*^20^*,* reported that sea level rise and associated tidal incursion are the key factors in sinking of the Munroe Island. He listed the various reasons, including the construction of the *Thenmala* dam in the upper course of the Kallada river, the Tsunami of 2004, tectonic activities and the choking of drainage network in the island etc. are as the causative factors for the environmental degradation of the Munroe Island. According to Jha et al.^21^, the sinking phenomenon of Munroe Island is attributed by rising sea level, tidal ingress, piping and erosion, paleochannels, organic matter, and the absence of deposition fluvial deposits. The Human Empowerment & Livelihood Promotion (HELP) Foundation^22^, in their quarterly report of 2016–2017, reported that the damming of Kallada river is the main reason for the sinking of Munroe Island. Saranya^23^ reported the factors reasonable for the sinking of Munroe Island are tidal effects, plate movement due to tsunamis, dam construction and mining activities. Reji Joseph^24^ reported about the environmental issues faced by the Munroe Island and he assumed it as a vanishing piece of land. Vincent et al.^25^ used interferometry techniques to study the subsidence/submergence of Munroe Island and confirmed the perceptible sinking of buildings and linked it with about the possibilities of land subsidence.

All the above researchers studied the estuarine island issues around the world were concentrated on some specific parameters and their analysis describes the causative factors of the land degradation. Some of them addressed the vulnerability assessment and the others were concentrated on environmental problems and the associated risk factors. There is no found any scholars addressed the land degradation issues of the estuarine island to understand the geomorphological disturbance and the natural and human influences caused to such disturbances. It is not possible to limit the driving forces of any geomorphological imbalance on earth surface to a limited extend. Assessing the geomorphic evolution and transformation of the disturbed landscape is very essential to frame out a sustainable management plan fulfilling the Sustainable Development Goals of United Nations formulated by its member states to better the world sustainably. This study adopts a multidisciplinary approach in order to understand the various environmental issues and its causes for the degradation of Munroe Island and proposes suitable management strategies for the proper conversation of this estuarine island ecosystem.

## Study area

**Figure 2:**
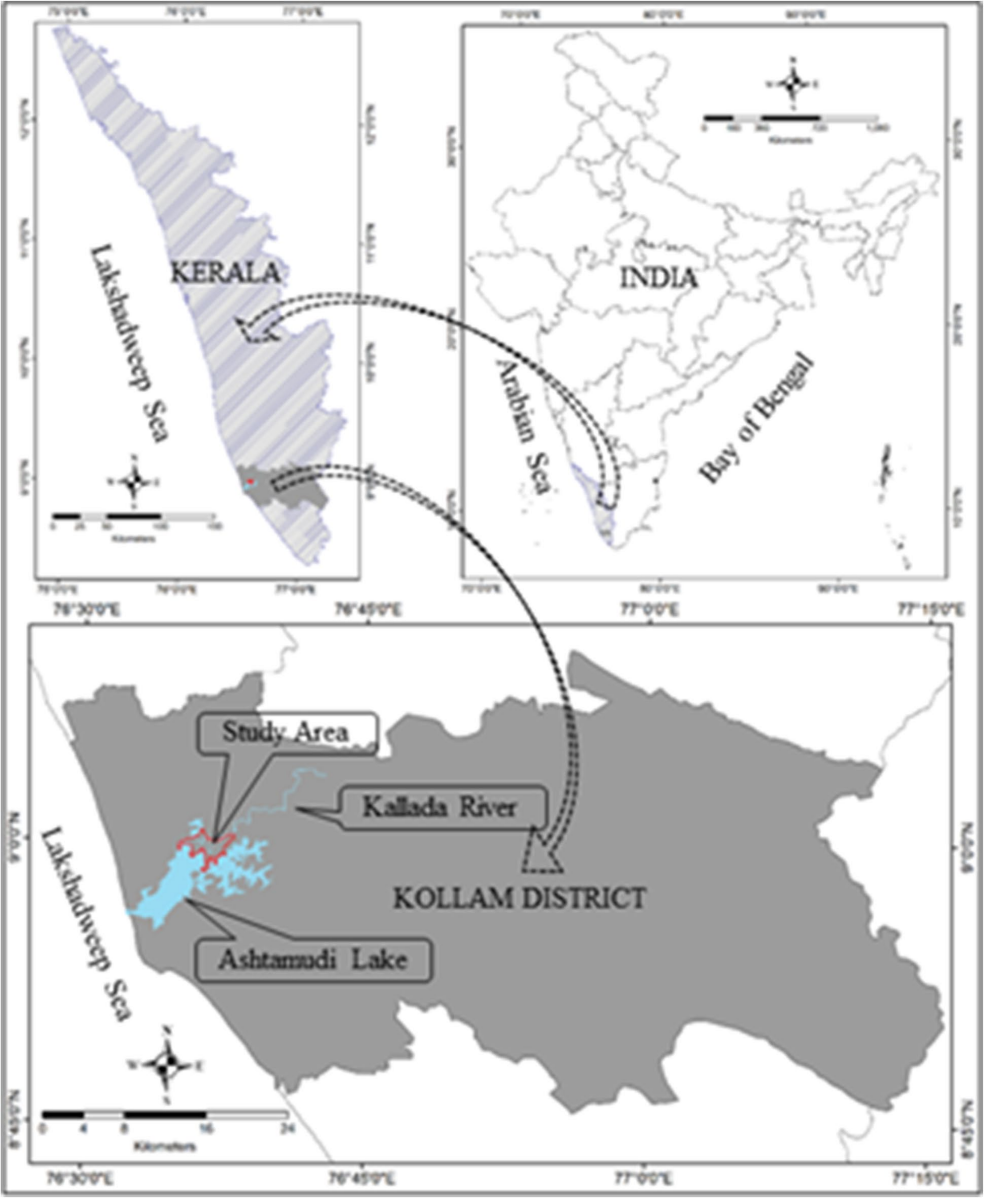
Location map of the study area (*Mundrothuruth GP,* Ashtamudi lake and Kallada river) (figure was generated by Arc GIS 10.6).

Munroe Island, locally known as *Mundrothuruth*, is a Gramapanchayat (GP) in the Kollam district of Kerala state and located on the southwest coastal plain of peninsular India (Fig. 2). The administrative boundary of the *Mundrothuruth* GP comprises the estuarine islands of the Ashtamudi backwater and surrounding flood plains of the Kallada river. To address the environmental degradation of the estuarine island ecosystem of the *Mundrothuruth GP*, the adjoining Ashtamudi backwater and Kallada river were also included in this study for understanding the geomorphological influence of the surrounding waterbody. With an area of 13,400 Hec (134 km^2^), this panchayat consists of 8 major islands and several small islets formed as estuarine islands at the conjunction of the Kallada river and Ashtamudi Lake. The boundaries of these island groups are well defined by Ashtamudi lake in the south and west, the flood plains of the Kallada river in the north and east, separated by natural streams from the mainland. There is an artificial river of 50 m wide, named as *Puthanar,* connecting the Kallada river and *Kanjirakode* lake of the eastern Ashtamudi backwater. This distributary of the Kallada river was constructed 100 years back for regulating the flooding and over sedimentation by splitting the river course into west and south arms.

### Geomorphological characteristics

**Figure 3:**
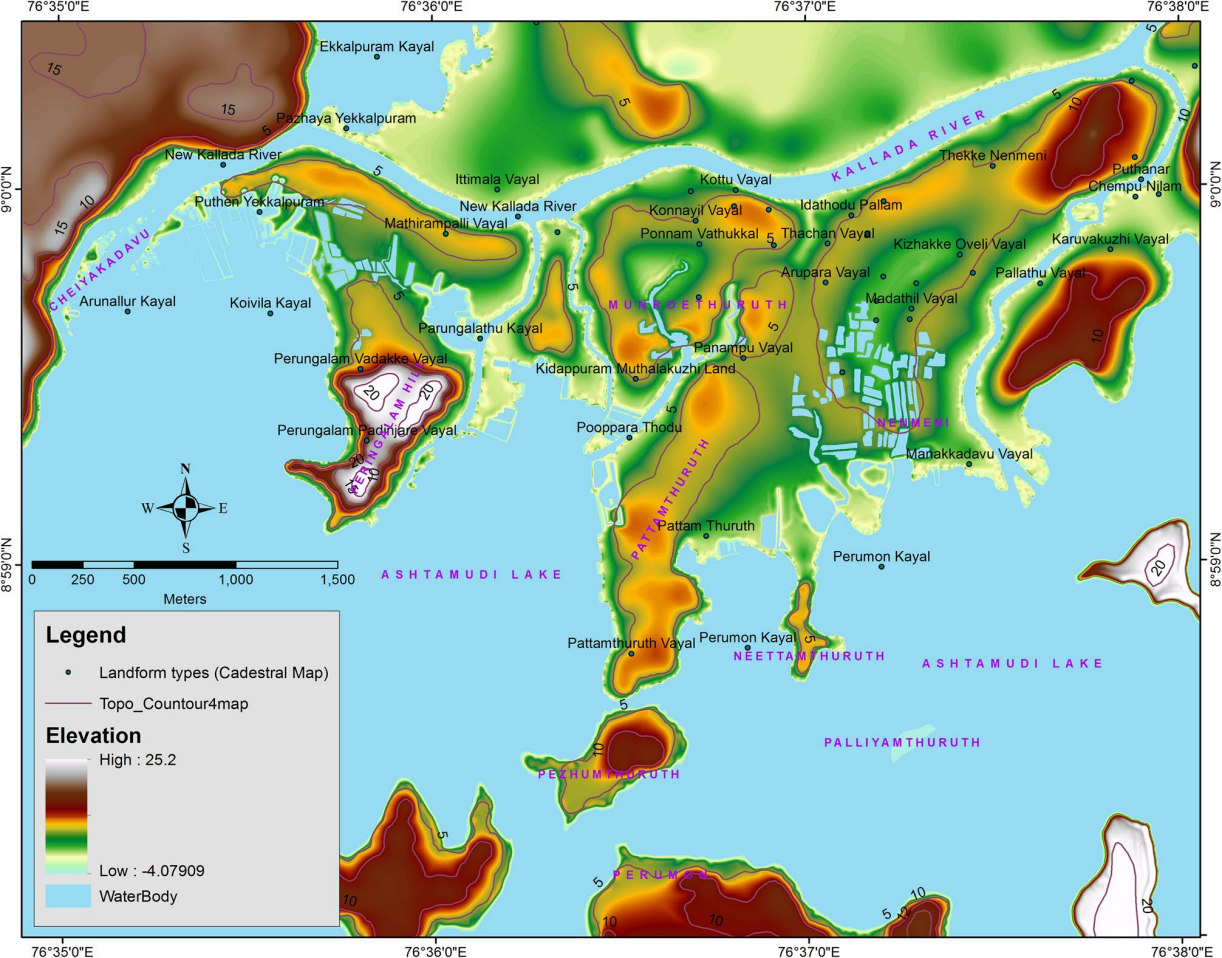
DEM of the study area prepared in RS & GIS platform; Contour lines and landform types are also marked [*Source: Cadastral Map of Revenue Department, GoK & Topographical Maps, SoI* (Figure was generated by Arc GIS 10.6 & ERDAS IMAGINE 2015)].

The Munroe Island are composed of newly formed alluvium brought up by combined riverine and marine sedimentation of Quaternary origin. Fluvio-marine forces of the Kallada river and Ashtamudi estuary modulate the geomorphic processes of Munroe Island. The Kallada river originates from the *Karimalai* hills of Western Ghats at 1524 m above MSL^26^ and discharges into Ashtamudi backwater at Munroe Island after flowing 121 km. Due to the high elevation gradient (average: 12.6 m/km) of the Kallada river, a considerable volume of sediments brought from the Western Ghats was deposited at the river in conjunction with the Ashtamudi backwater^26^. Alluvium of Holocene formation was deposited at the valley of a few residual hills of Tertiary (lateritic) formation, such as *Peringalam, Pezhumthuruth, Pattamthuruth, Neettamthuruth*, etc. Geomorphologically, these island groups may be classified as denudational hills, valley regions, flood plains and Filtration ponds (Wetlands) (Fig. 3). About 58% of the land area of the Munroe Island is distributed at a height of 5 m above MSL (Survey of India Topographical sheet), and the highest point is having an elevation of 21.8 m AMSL, which is at *Peringalam* hill. Several islands and islets formed due to the drainage networks of natural and artificial streams connecting the different waterbodies at Munroe Island.

### Geology of the area

The Munroe Island area is a part of the Ashtamudi estuary, which forms a crucial geological segment of the South Indian peninsular shield; both crystalline rocks and tertiary sediments are significant components of the estuary^21,27^. Geologically, this stretch of land has been referred to as the South Kerala sedimentary basin (SKSB) by Nair et al.^28–30^. Stratigraphically, the area comprises three principal rock formations: the Archaean crystalline basement and tertiary and quaternary sedimentary sequences^30^. The Quaternary deposits are represented by alluvial clays, sandy clays, and peat on the lake's southeastern side. Most of the geological studies of Munroe Island reveal that the Quaternary deposits' sediment thickness is up to 25 m^31,32^. The Precambrian crystallines and Neogene and Quaternary sediments occupies the western part of the study area. The Neogene sediments are part of two prominent formations viz. Quilon and Warkalli Formations of Lower Miocene age^33,34^. The Warkalli Formation represents sandstones and clays with intercalations of lignite seams at the base and beyond the Warkallis, and fossiliferous limestones and sandy carbonaceous clays are composed as part of the Quilon formation. The landward extension of the offshore sedimentary basin between Kollam and Kodungallur of SW peninsular India, with a sediment fill of ca. 700 m thickness, is referred to as the South Kerala Sedimentary Basin (SKSB) by Nair et al.^28–30^ and Padmalal et al.^32,33,35^ by considering the uniqueness of the entire peninsular west coast. Morphotectonics and sea-level oscillation studies of the coastal tracts of the southwestern coast of India reveal that a significant part of the SKSB is in a submerged block. Cliffs and bays characterize areas in the southern and northern regions, indicating emergence and subsequent coastal erosion under rising sea levels in the Late Quaternary^32,36,37^. Ashtamudi is situated in this uplift block, and the estuary shows antecedent characteristics^31,33^.

### Socioeconomic parameters

**Figure 4:**
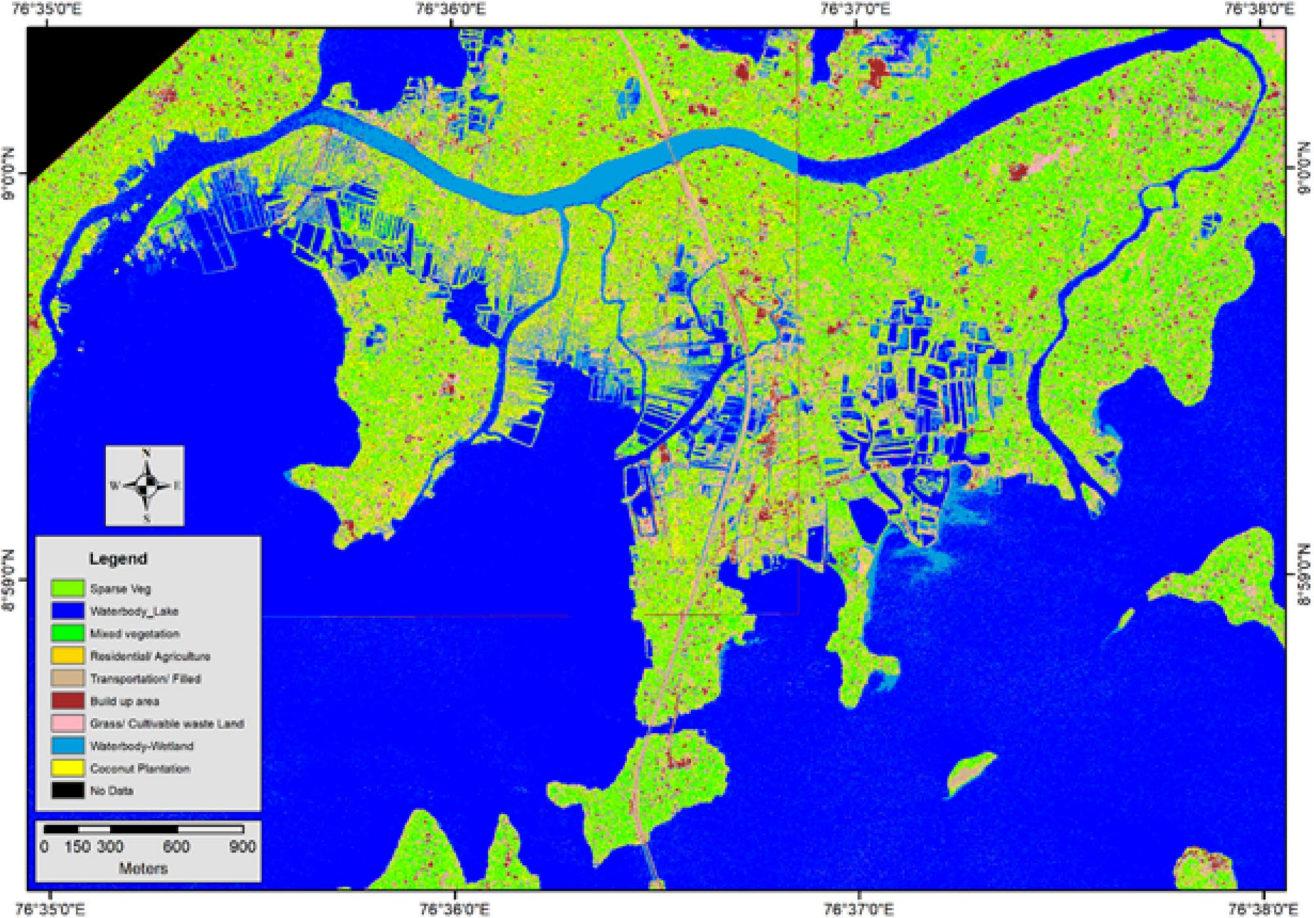
Land use pattern—supervised classification of World View—II images (figure was generated by Arc GIS 10.6 & ERDAS IMAGINE 2015).

*Mundrothuruth* GP, having a population of 9440 persons reside in 2505 houses as per the 2011 census^38^, is only one Gramapanchayat or local body of Kerala where no PWD (Public Work Department, Govt of Kerala) roads. As per the annual report of *Mundrothuruth* panchayat^39^, 18.75 km long footpaths and 4.27 km metaled roads are present here. The *Idiyakkadavu* bridge is the only one-road entry in to this Gramapanchayat. Two ferry services and many small boat services serve the transportation needs of the inhabitants. The 3.36 km long railway line, passing through the island's heart, splits the island into the easily accessible east and less accessible west sides (Fig. 4). The western part of this railway line is an underdeveloped region and does not have any better transportation facilities except waterways.

Rice cultivation was the principal economic activity of the inhabitants in the 1950s and now local people are more dependent on coconut cultivation and tourism for their livelihoods. There were about 227 acres of paddy fields in 1950, and considerably reduced to 8 acres in 1995 and less than 2 acres in 2018^39^. After the construction of the Thenmala reservoir in the upper stream of the Kallada river there is a decrease in the supply of fresh alluvium during the rainy season and the rice cultivation was critically affected, so the farmers started to plant coconut trees, as it offers better yield and economic value. Due to the increased salinity of the water and soil and the lack of fresh alluvium to manure plantations, now the coconut cultivation is also under crisis.

## Methodology

The available remote sensing data and land survey records were analyzed to understand the Munroe Island’s morphological changes during the last six decades. Considering the constraints of the scale factor of available secondary datasets for such a small area, the aerial photographs of 1989 from the National Remote Sensing Centre (NRSC), Hyderabad, (1:15,000) were taken for the preparation of base map. The planimetric cadastral survey sheets of 1964 prepared in 1:3990 from the Department of Survey & Land Records, Kerala were used to interpret the land features of 1950–1960. After resampling to a better resolution, different satellite images, such as Landsat, World View II, Sentinel and CARTOSAT, were also used. The details of the secondary data sets benefitted for the study are given in Table 1. Erdas Imagine 2015 and Arc GIS 2015 were used to integrate the available datasets into a georeferenced standard grid system for further analysis. The high-precision GNSS receivers are combined in static mode to obtain ground control points with sub-centimeter accuracy.

**Table 1:** Different data sets used for the geomorphological studies.

Sl. No.	Data type/source	Publisher	Year of survey	Scale/resolution
1	Cadastral/village maps	Revenue Department–Government of Kerala	1950–60	1:3960
2	Topographic sheet	Survey of India	1977–78	1:25,000
3	Aerial photographs	National Remote Sensing Centre (NRSC), Hyderabad	1989	1:15,000
5	Imagery/Landsat 7	Global Land Cover Facility	2000	15 m
8	Imagery/World View-2	Digital Globe	2011	0.5 m
9	Imagery/CARTOSAT	NRSC	2014	2.5 m
10	Imagery/Sentinel 2	Copernicus Open Access	2021	10 m

Electrical resistivity meter surveys were conducted to understand the sub-surface geology of the land area. Multichannel Electrical Resistivity (ER) imaging is a scientific and reliable tool for interpreting subsurface lithological features especially the Quaternary deposits and it is also used for coastal groundwater studies, as subtle shifts in the fresh water/saltwater interface can be readily visualized using standard inversion routines^40,41^. Schlumberger, Wenner Alpha and Dipole‒Dipole experiments were repeated for the exact location to confirm the area's lithology. The soil investigation reports prepared through the SPT drilling method by the Public Works Department for the different locations within the island area were collected and analyzed to understand the geological structure of the island. The present bathymetric status of the Kallada river and Ashtamudi backwater system was surveyed using an echo-sounder (Ceeducer Pro). Historical information about the nature of river and backwater systems was collected from the Island inhabitants through Focus Group Discussions and personal surveys. The representatives of different social categories of the Island inhabitants were formed into separate Focus Groups and refined the field information to utmost errorless. The results of the present study were cross verified with the information collected from such discussions and interviews.

## Analysis and discussions

Land vulnerability of an area is directly related to the natural as well as anthropogenic activities involved in the geomorphological unit. Being one of the most vulnerable ecosystems, the estuaries and estuarine islands are delicately affected by both ecological processes of the sea and land and have pressures from multiple anthropogenic stressors and global climate change^42–44^. Ecological vulnerability and ecological sensitivity are similar and both originated from the concept of ecotone^10,45^. The geomorphologic concept of landscape sensitivity was first proposed by Brunsden and Thornes, who argued that the sensitivity indicated the propensity to change and the capacity to absorb the effects of disturbances^10,46,47^. Landscape sensitivity is studied by many researchers such as Allison and Thomas, Miles et al., Harvey, Knox, Usher, Haara et al., Thomas, Jennings and Yuan Chi^8,47–54^, through different case studies. Based on their findings Yuan Chi summarized the important characteristics of the landscape sensitivity are: a, the change of the landscape ecosystem; it involves the change likelihood, ratio, and component, as well as the resistance and susceptibility to the change, b, the temporal and spatial scales; which determine the occurrence, degree, and distribution of the change, c, the external disturbances that cause the change; the disturbances included natural and anthropogenic origins with different categories and intensities, and d, the threshold of the landscape sensitivity; it refers to the point of transition for the landscape ecosystem^8^. The environmental vulnerability of the Munroe Island has been studied based on the characterization of the geomorphological and sociocultural dynamics of the region based on the above characteristics.

### Bathymetric surveys in Ashtamudi lake and the Kallada river

**Figure 5:**
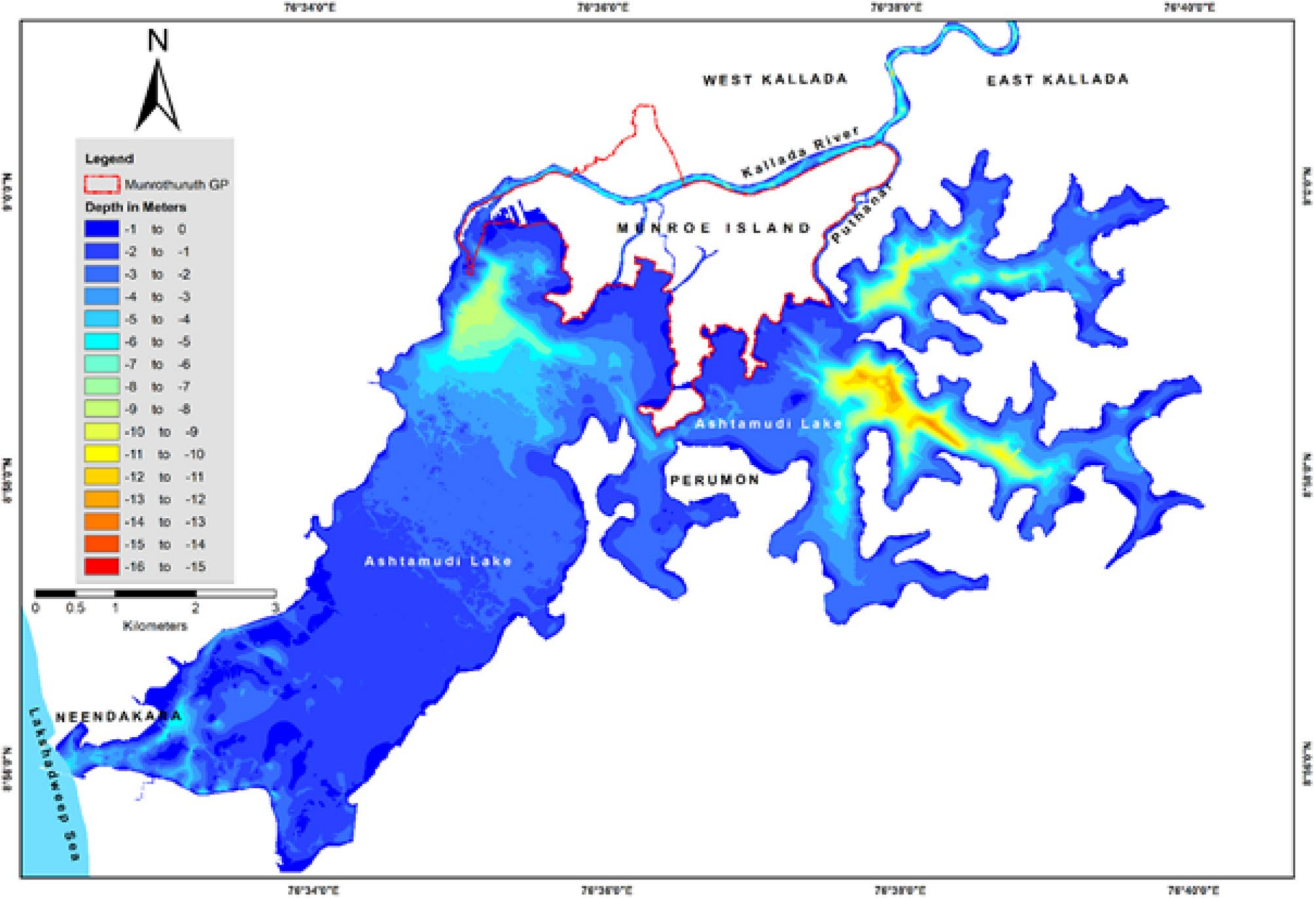
Bathymetric profile of Ashtamudi lake and adjoining Kallada river (Figure was generated by Arc GIS 10.6).

The present study shows that the geomorphic processes occurring on the Munroe Island are affected by anthropogenic disturbances in the morpho-dynamics of the Kallada river, Ashtamudi backwaters and associated fluvio-tidal interactions. A detailed bathymetric survey of both water bodies up to the tidal-influenced upper limit of the Kallada river^27^ was conducted with 200 m spaced grid references (Fig. 5). Bathymetry shows that the deepest point of the Ashtamudi backwater system is in *Vellimon* lake (13.45 m), the SE extension of Ashtamudi lake. The eastern side of Ashtamudi lake is deeper than the western side of this backwater system. The depth of the backwater decreases towards the estuary, and most parts of the lakebed are exposed here at the mouth of the inlet during the low tide. Compared to Ashtamudi lake, the Kallada river is deeper, and the riverbed area is recorded as the average depth is greater than 13 m. The deepest part of 14.9 m is recorded near *Kunnathoor* bridge, which is 12 km upstream from Munroe Island. Except for a few spots of hard (resistant) rocks, the river fairly and consistently follows a higher depth throughout its course.

Once the Kallada river supplied very fertile alluvium during its flooding seasons (monsoon/rainy season), and most of this alluvium is deposited in the floodplains of the Munroe Island and the Ashtamudi lake. With a vast river catchment area from elevated lands of Western Ghats and a shorter course of 121 km^33,55^ and a higher elevation gradient of 12.6 m/km^56^, the Kallada river has a higher transporting capacity. The eroded surface and mined river/lakebeds at lower courses were replaced by the sediment load supplied by the Kallada river during each flood season until dam construction. During the focus group discussions with residents of the Island, they had described that they were crossing the Kallada river on foot in the 1990s or even earlier during the dry seasons. The construction of the Thenmala reservoir dam in 1980s across the river drastically choked the sediment supply of the Kallada river. In addition, excessive commercial sand mining without any regulation from the riverbeds of Kallada and Ashtamudi waterbodies accelerated the deepening of waterbodies. It increased the erosion of surface and subsurface soils through fluvial and hydraulic action. This, in turn, drastically reduced the deposition of fertile alluvium over the low-lying Munroe Island. The current bathymetry shows that the river channel has deepened its course to 14 m compared to 5–6 m of 1980s. When comparing the bathymetric data of 2001^27^, it is interesting to note that no considerable changes occurred in the bathymetry of Ashtamudi lake over the last two decades.

Dams indeed alter aquatic ecology and river hydrology, upstream and downstream, affecting water quality, quantity, breeding grounds and habitation^22^. The other significant impact of the damming of the Kallada river is the saline water intrusion towards upstream of Ashtamudi lake and the Kallada river. The freshwater discharge is regulated after the construction of the Thenmala reservoir, and the water is being diverted to the reservoir and associated canals. There is a decline in sedimentation over the floodplains and catchment area as a result of the increased tidal effects and associated running water dynamics, which may accelerate the erosion trend of the nearby places.

### Lithological characterization of the Munroe Island

**Figure 6:**
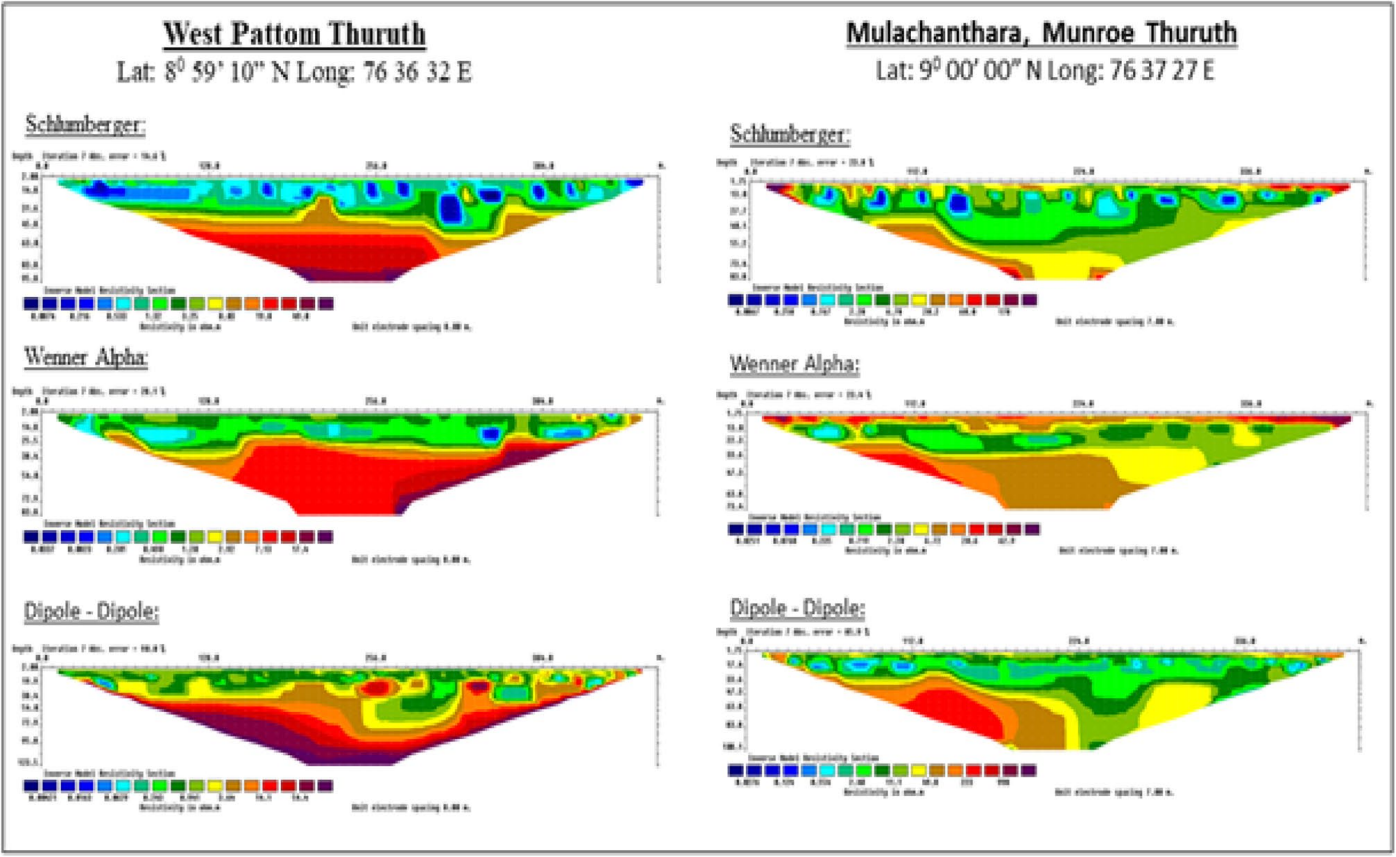
Electrical resistivity profiles of Munroe Island.

**Figure 7:**
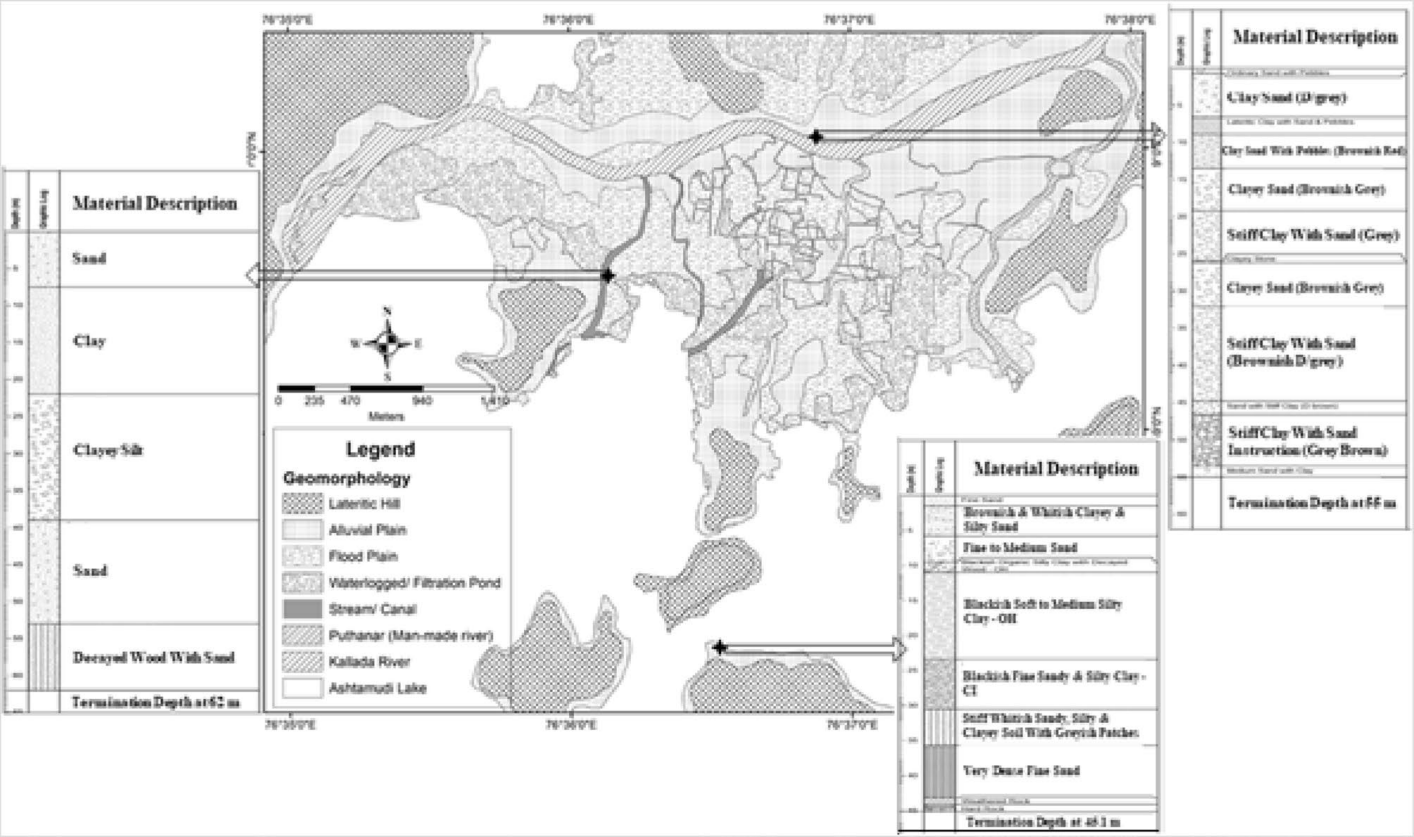
Geomorphological map showing litho-log of north (*Kannamkadu*); middle (*Konnayil Kadavu*); and south (*Perumon* bridge) locations of Munroe Island *(borehole data source: PWD, Govt of Kerala)* (Software used: Arc GIS 10.6).

The Munroe Island is a riverine delta formation by the Kallada river at the conjunction of river and backwater systems. To understand the micro-geomorphological processes of the study area, the near-surface geology of the Munroe Island had been studied in detail with the help of resistivity meter surveys and borehole datalogs from different locations. As per the current resistivity survey, it is evident that the Munroe Island is formed by recent unconsolidated loose sediments more than 120 m thick succession below ground level (Figs. 6 and 7). The electrical resistivity tomography of identified locations within the deltaic region shows a meagre resistance value to its maximum penetration (Fig. 6), which proves that the sedimentary column with intercalations of sand and carbonaceous clays of varying thickness extends to a depth of 120 m, in turn indicating the process of enormous sedimentation happened during the recent geological period. Loose wet soils of saline nature records a lower resistance value for an electric circuit. The layers formed in the diagram (Fig. 6) represent the seasonal deposition of unconsolidated soils as thin sequence. The *Mulachanthara* station of the resistivity meter tomography, which is situated at a more stable location of the Island, has a higher resistivity value than the West *Pattamthuruth* location, which is located at the exact alluvial flood plain.

The Public Works Department (PWD), Kerala State carried out soil profile studies through Soil Penetrating Test (SPT) borehole drilling method as part of constructing bridges at three different locations up to a depth of 62 m, i.e., one across the Kallada river (north side)^57^, one across Ashtamudi lake in southern Munroe Island^58^ and one at the central part of Munroe Island (across a canal)^59^ (Fig. 7). The hard rock is found only on the southern side of the lake at a depth of 45 m. The litho-log shows that unconsolidated loose sediments of significantly higher thickness occur in the entire Munroe Island (Fig. 7). Anidas Khan et al*.*^60^ studied the shear strength and compressibility characteristics of Munroe Island's soil for two different locations with disturbed and undisturbed samples. They classified the soil of *Mundrothuruth* into medium compressibility clay (CI) and high compressibility clay (CH) with natural moisture contents of 44.5% and 74%, respectively. The unconfined compressive strengths of the undisturbed and remolded samples for the first location are 34.5 kN/m^2^ and 22.1 kN/m^2^, respectively, while they are 13 kN/m^2^ and 9 kN/m^2^ respectively for the second location^60^. Such compressive strength indicates that the soils of Munroe Island are soft or very soft in nature.

### Land degradation: a morphological analysis

**Figure 8:**
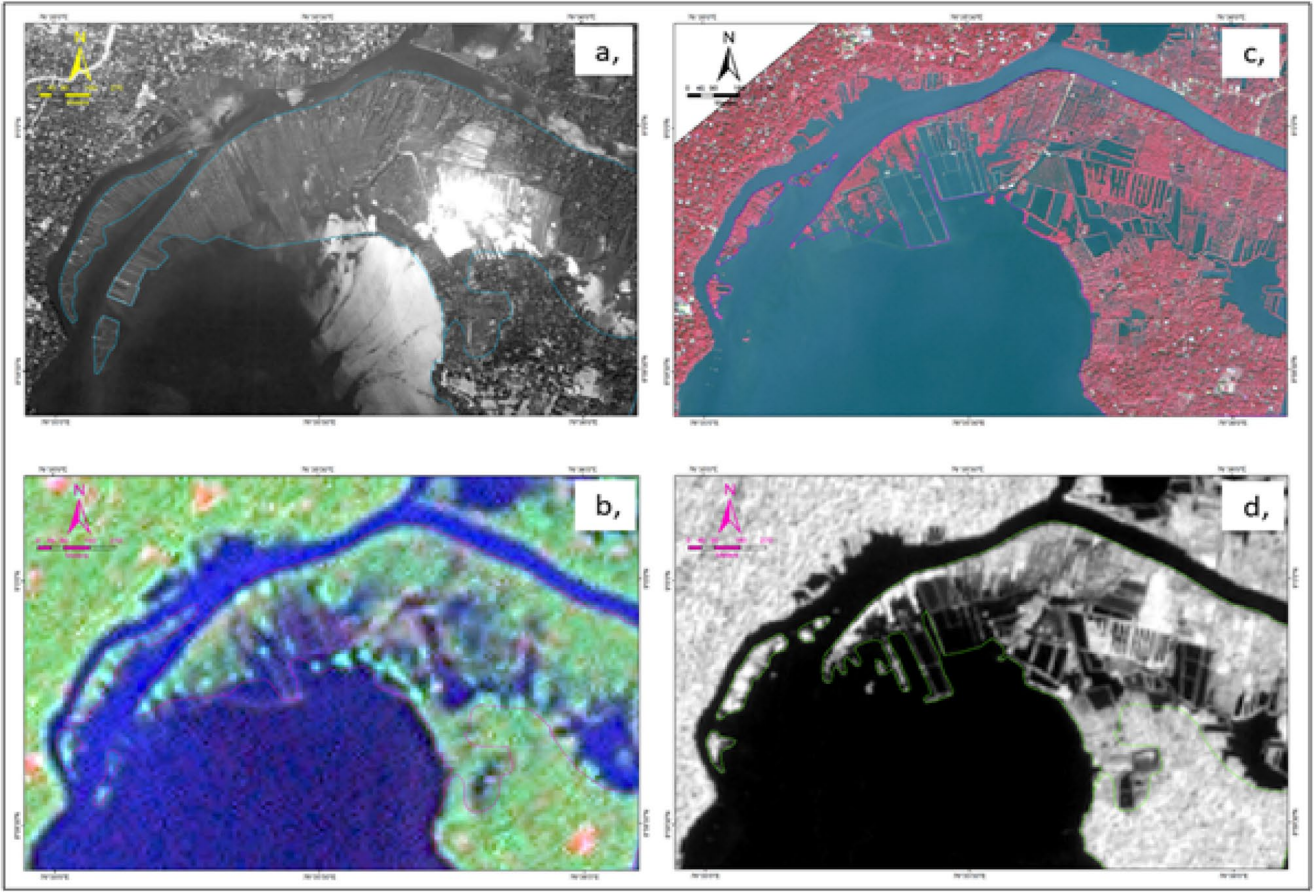
Morphological changes in the study area from the satellite images (**a**) 1989 (aerial photograph); (**b**) 2000 (Landsat); (**c**) 2011 (World View—II); (**d**) 2021 (Sentinel) (the modified maps of (**a**) is obtained from National remote Sensing Centre (NRSC), Hyderabad, (**b**) is downloaded from https://earthexplorer.usgs.gov/ (**c**) is obtained from Digital Globe through NRSC and (**d**) is downloaded from https://scihub.copernicus.eu/. Figures were generated using Arc GIS 10.6).

**Figure 9:**
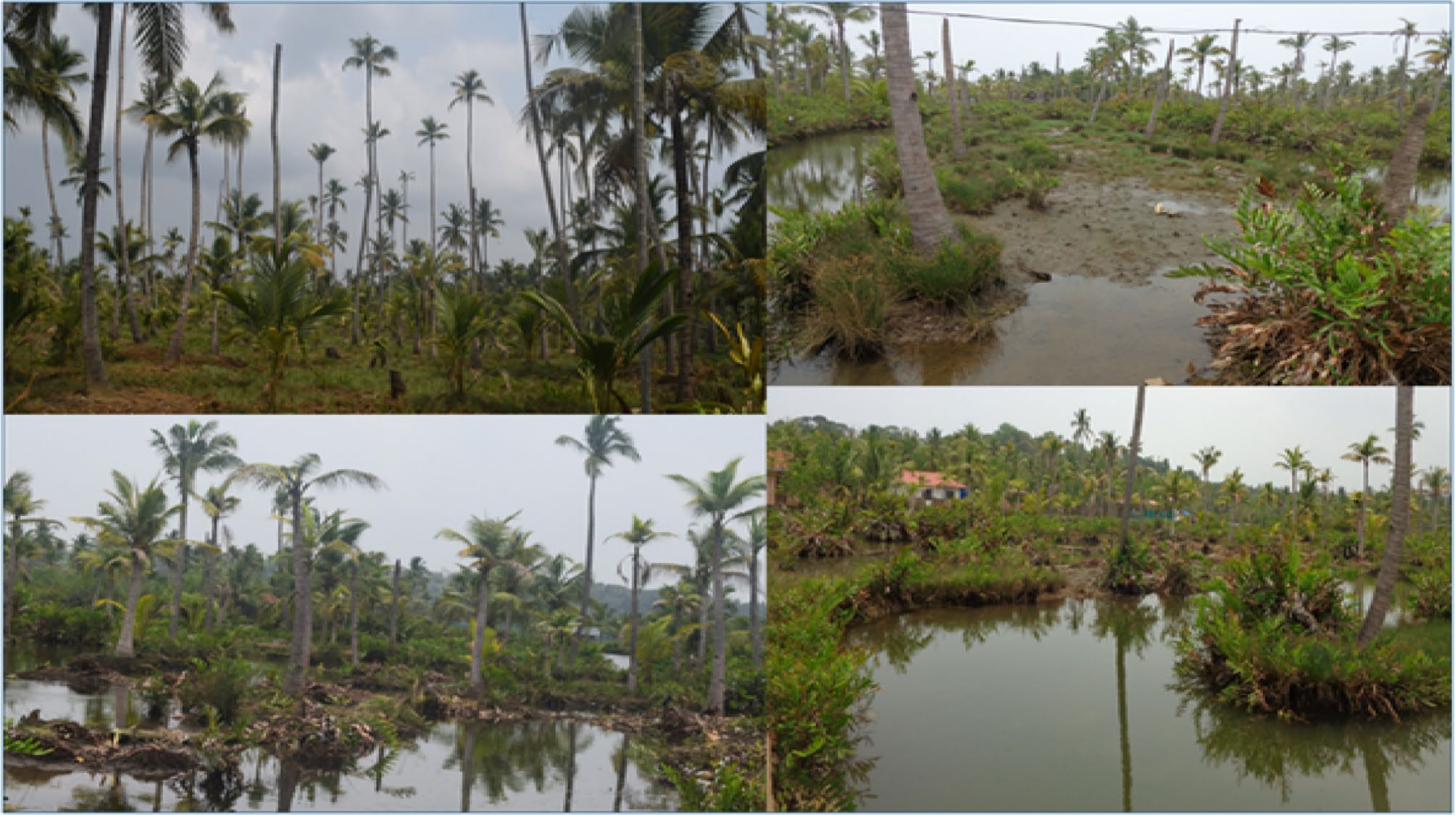
Threatened coconut plantations indicating the low productive regime. Photographs taken by Rafeeque MK.

To decrease the impact of the monsoon floods and to distribute the alluvium to the southern part of the island, Canol Munroe, the then Diwan of the Thiruvithamkoor Dynasty, made an artificial man-made canal during the 1820s connecting the Kallada river with the eastern extension of Ashtamudi lake, and this river is known as “*Puthanar*” (meaning a new river). During the last few decades, (after 1980s) the estuarine island ecosystem of Munroe Island has faced several structural deformities. The natural sedimentation and flooding happening in the Islands were very limited and hence, the normal events happened over the past several decades disturbed and significantly affected the land neutrality. These islands, once known as the region's rice bowl, now devoid of any paddy cultivation mainly because of the increased soil salinity. According to the Cadastral map prepared by the revenue department (1960s) there were many paddy fields, locally named as *Mathirampalli Vayal* (Vayal is the local name for paddy field), *Thekke Kothapppalam Vayal, Mattil Vayal, Kottuvayal, pallaykattu Vayal, Konnayil Vayal, Vadakke Kundara Vayal, Thachan Vayal, Thekke Kundara Vayal, Kizhakke Oveli Vayal, Thekke Oveli Vayal, Odiyil Vettukattu Vayal, Nedumala Vayal, Madathil Vayal, Karichal Vayal, Moonumukkil Vayal, Arupara Vayal, Kaniyampalli Vayal, Manakkadavu Vayal, Panampu Vayal, Pattamthuruth Vayal* etc. The recent satellite images shows that no paddy cultivation exist now, which is further confirmed by the field observations conducted through our study. The annual report published by Gramapanchayat^39^ indicate that the paddy field of region was reduced from 227 to 8 acres (from 1950 to 1995) and now about in 2 acres only (2018). Most of the paddy fields of northern and northwestern regions are severely affected by land degradation due to erosion, saline water intrusion and flooding and are entirely or partially buried under the backwater system. Figure 8 depicts the morphological degradation of the severely affected areas of Munroe Island from 1989 to 2021 through different satellite images. Some paddy fields are converted into filtration ponds to take the benefit of frequent tidal flooding. The coconut plantations were later introduced in place of paddy fields, and they eventually replaced the paddy fields. However, during the last decades, it has been observed that these coconut plantations are also under threat mainly because of degradation of the soil fertility, which directly bears the quality and quantity of production (Fig. 9).

**Figure 10:**
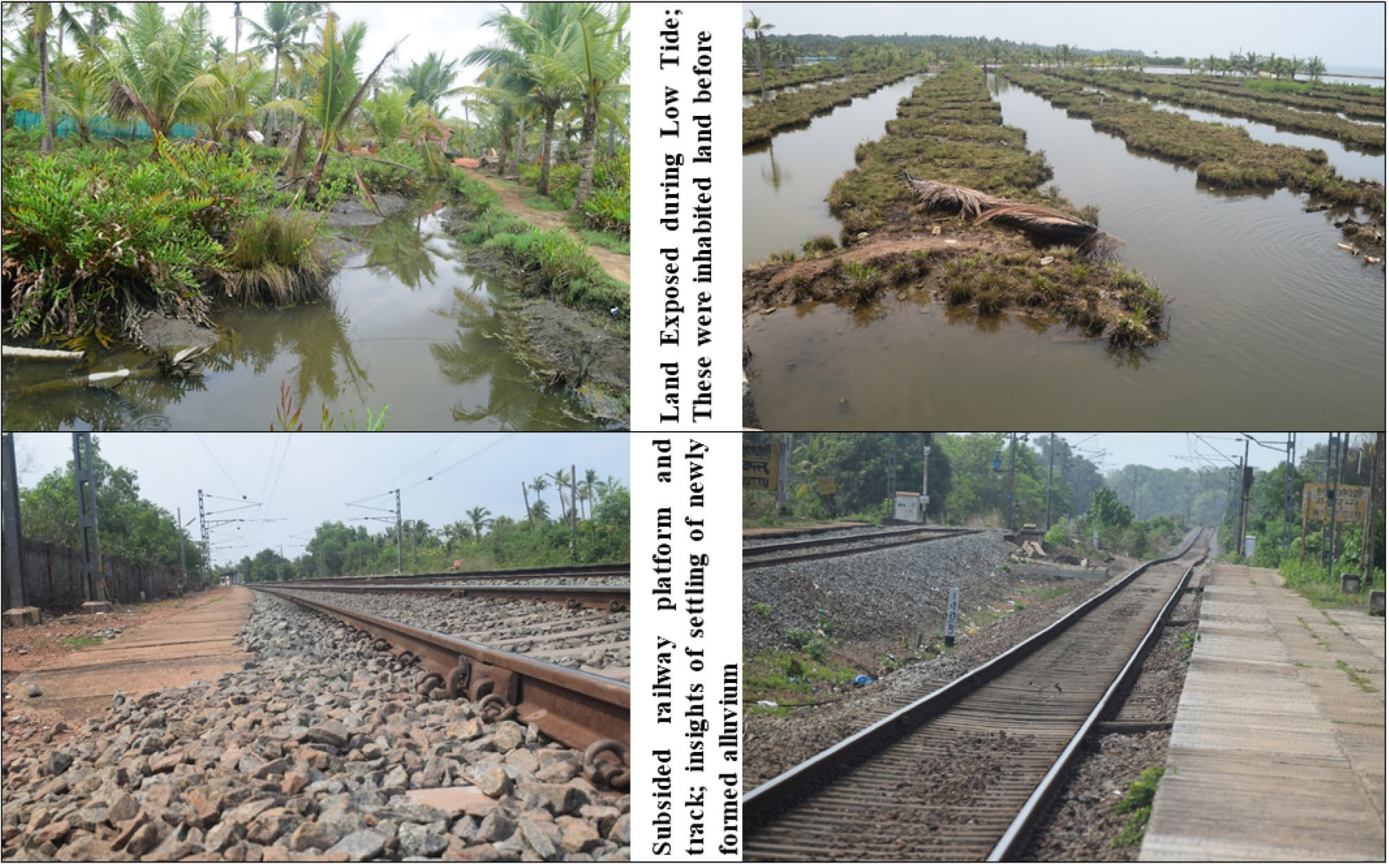
Various environmental degradations in Munroe Island. Photographs taken by Rafeeque MK.

Over the study area the most affected alluvial plain of the *Peringalam* and *Cheriyakadavu* island are taken separately to study the morphological changes over the decades. This area is named *Puthan Yekkalpuram* (which means new alluvium land), and the north side of the Kallada river (the northward extension in the *Mundrothuruth* GP) is demarcated as old alluvium land (*Pazhaya Yekkalpuram*) as per the revenue department’s cadastral map. The study shows that total 38.73 acres of land has lost from the *Peringalam* and *Cheriyakadavu* Islands during the last 32 years, which is equivalent to 11.78% and 46.95% of the total geographical area of the *Peringalam* and *Cheriyakadavu* Islands, respectively. The land degradation details over the last three decades are given in the Table 2. Many other locations, such as *Nenmeni* and West *Pattamthuruth*, are also severely affected by land degradation. However, these areas are landlocked and less affected by running water or floods. Hence, the land degradation experienced is the settling of the topsoil and subsidence of structures such as houses and bridges. The sinking of basements of many houses and even the subsidence of railway platforms are well observed during field visits, indicating the alarming land degradation issues (Figs. 1 and 10) to be addressed its deserving importance. There are also clear indications of the gradual formation of new waterlogged areas in the islands, which may further deteriorate and forms the part of the backwater system which eventually affects total land area of the Munroe Island.

**Table 2:** Land degradation of *Peringalam* and *Cheriyakadavu* region for the past 32 years.

Year	Peringalam alluvial plain			Cheriyakadavu island		
	Total area (acre)	Decadal land degradation (acre)	Decadal land degradation (%)	Total area (acre)	Decadal land degradation (acre)	Decadal land degradation (%)
1989	272.66			14.08		
2000	260.02	12.63	4.86	11.63	2.45	21.04
2011	247.07	12.95	5.24	8.68	2.95	34.04
2021	240.54	6.54	2.72	7.47	1.21	16.19
Total		32.12	11.78		6.61	46.95

The island population also shows a negative growth over the years. According to the census report of 2011^38^, the total population of Gramapanchayat has decreased to 9440 person/km^2^ in 2011 from 10,013 person/km^2^ of 2001 and 10,010 person/km^2^ of 1991 census reports. Frequent flooding (especially tidal flooding), the lack of drinking water, and migration in search of a better livelihood are the main reasons for the observed population reduction as revealed through the survey. The high intrusion of saline water into the cultivated land through tidal flooding and the lack of flushing of surface saline soils by monsoon floods (freshwater) decreased agricultural productivity of the area, and hence, now people are more dependent on fishing and backwater activities for their livelihood. Lack of proper transportation to the nearby markets limits their fishing activities to a daily subsistence level. Due to the flooding caused by subsidence/tidal surges and land degradation during the last few decades, more than 500 households have vacated their houses^38,39^.

### Tidal Flooding and Estuarine Processes

In Mundrothuruth, the major environmental degradation problems where occurring due to tidal flooding and saline water intrusion into the freshwater ecosystem. Mathew et al. studied the tidal and current mechanisms of the Ashtamudi backwater in 2001^61^. They reported that the Kallada river plays a vital role in determining the eastern lake's circulation pattern. In addition, the increased discharge from the north Chavara canal and the south Kollam canal also influences the local circulation of the Ashtamudi backwater. The current velocity reaches up to 100 cm/s at the estuary entrance, but it rapidly diminishes in the eastern parts, where the speed is generally less than 30 cm/s. One of the critical observations made during the field study, which corroborates with the acquaintance of local people as well, is that the flooding on Munroe Island is not related to the spring tide of the open ocean. The disappearance of the semidiurnal tide in the central lakes occurs due to frictional resistance and the time lags for the tide to travel across the estuary^61^. At the shorter semidiurnal period of approximately 12 h, the tide is more dissipated than the more extended constituents of 24-h duration. The survey conducted with the island inhabitants also reiterates these views.

**Figure 11:**
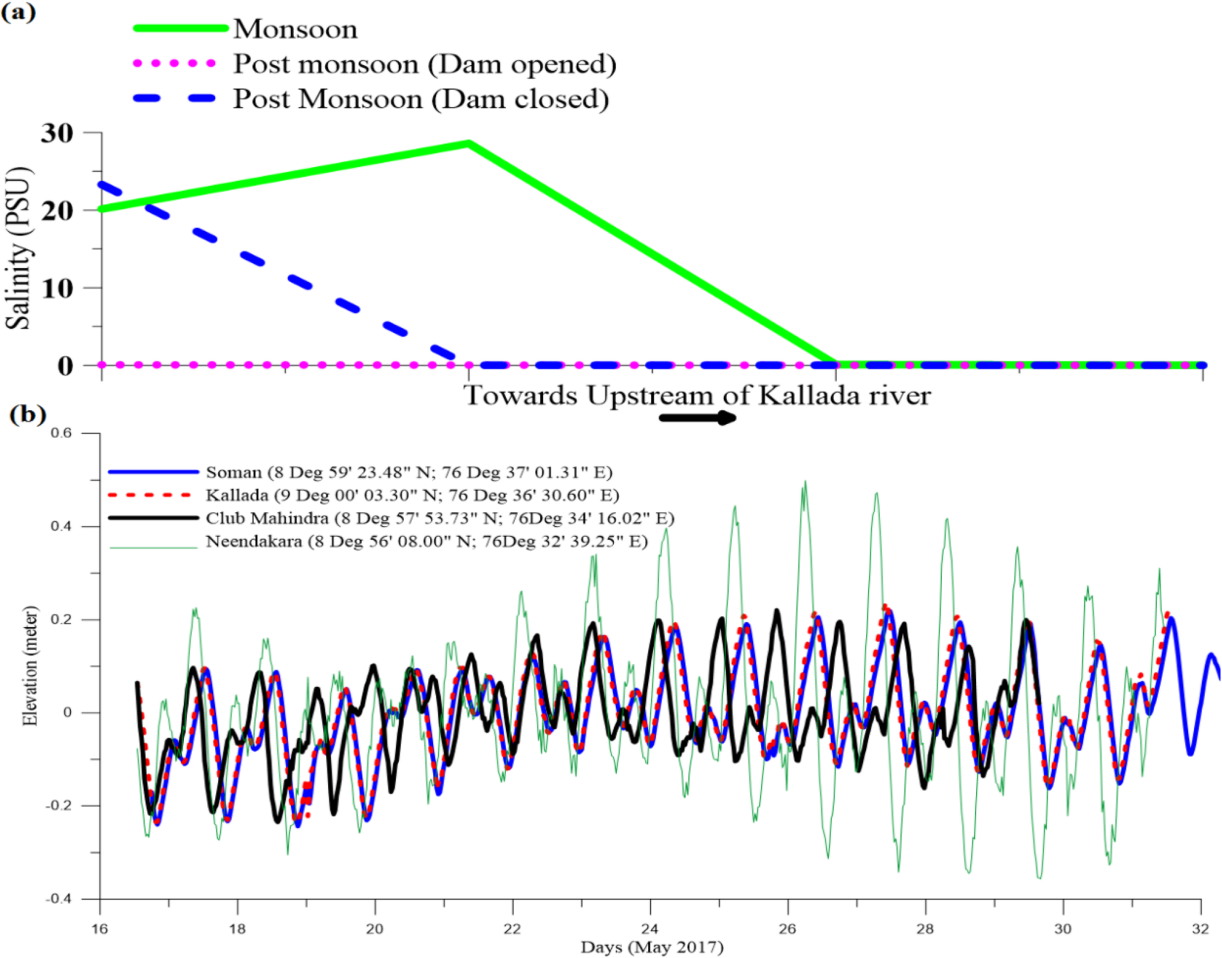
(**a**) Salinity variation of bottom water at selected locations in Kallada river during monsoon and post monsoon. (**b**) Observed tide during pre-monsoon months.

As per the experience of local inhabitants, tidal flooding in Munroe Island was not frequent in earlier times. The comparison of the bathymetry data collected during 2000^58^ and 2017 (Fig. 5) in and around the regions of Munro Islands shows that there is not much change in bathymetry during the period. Hence, changes in basin geometry are not having a significant role in tidal dynamics in imparting the variations as observed. In addition to the bathymetric survey, the data on tide measurements at four locations corresponding to three seasons were also collected. The tide data measured during the pre-monsoon period is shown in Fig. 11a. The figure shows that the tidal range in the inland area is almost the same even during the spring and neap tides. As discussed earlier, the tidal flooding in Munro Island is not related to spring tide in the ocean, and there may be the influence of specific complicated dynamics in the basin for this flooding that needs to be studied more profoundly. Further the data pertaining to tidal dynamics were inadequate; we established three tide gauges in selected locations in and around Munro Island. From the analysis of tide gauge data, it is found that the signature of anomalous variability in water column height, which is not at all linked to the tidal dynamics.

The water quality analysis for three time periods, during the year of the cyclonic storm, Okhi (2017), was conducted to understand river run-up impact on salinity in and around Munroe Island (Fig. 11). The riverbed is lowered below the baseline of erosion, and dense saline water is trapped in the deeps during high tide. This has been confirmed during the bathymetric survey of the Kallada river and Ashtamudi backwaters, which showed a significant increase in water depth, particularly within the river channel. The high-density saline water is trapped in the basins and trenches created in the river channel due to uncontrolled sand mining, which leads to the degradation of the quality of sediments and groundwater in the region. Nevertheless, the samples collected immediately after *Okhi* (when the dam's shutter was opened due to heavy rainfall in the catchment area) show that the high runoff replaced the trapped saline water with fresh water. After ten days of the first sampling, the water became saline nature after the closure of the dam's shutter. This proves that because of dam construction, the river runoff in the Kallada river was reduced significantly, and extensive human interactions especially sand mining activities increased the riverbed deepening and formation of pools beyond the base level of running water.

### Conservations and management strategies

Considering the facts discussed above, the Munroe Island may continue to be badly affected unless suitable sustainable management strategies are not evolved. Construction and associated activities, such as the damming of reservoirs, sand mining and landfilling, are indispensable for any nation's economic and social development. United Nations's member states have formulated 17-point Sustainable Developmental Goals (SDGs) to better the world sustainably. Local and national governments pertaining to the Munroe Island need to develop a sustainable management plan to protect this Ramsar-listed wetland. The environmental issues of *Mundrothuruth* can be controlled, and land degradation may be monitored through a well-drafted working plan. All aspects of earth and social sciences may be integrated to draft such a management plan of reverse landscaping. The reverse landscaping (i.e., recalling the degrading landscape to its geomorphic isostatic state) method is a must-considered sustainable solution for land degradation and other environmental issues.

The deep courses of Kallada river must be upwarped through a well-planned artificial sedimentation to eradicate the saline banks of deep basins. The sediments deposited in the Thenmala reservoir and the sediments removed through the digging of boat channels may be utilized in a periodic monitoring method. Sand mining from Ashtamudi lake and the Kallada river may be strictly controlled, and the minimum freshwater flow should be ensured. The construction methods practiced in *Mundrothuruth* are outdated and technically nonexistent. Well-studied engineering methods suitable for an environmentally fragile area must be implemented with a proper understanding of the soil characteristics, such as shear strength and compressibility rate, and hydrodynamics, such as tidal and fluvial actions. Soil fertility must be increased by supplying additional fertile soil and freshwater, at least for a minimum period. The inhabitants' socioeconomic well-being is strengthened by advancing technology and providing easy access to the market and other social amenities.

## Conclusion

The monsoon floods once supplied plenty of fertile alluvium from the river catchments every year and hence contributed for a productive agricultural realm. The morphological studies based on satellite imageries indicate that after 1980s, the Munro Island is severely affected by agricultural productivity as well as land loss, which disturbed the land neutrality of the Island ecosystem. Though the degradation started in 1980s, the inhabitants experienced its bitter experiences around year 2000. The studies pertaining to the past three decades clearly revealed that almost 39% of the land area of the Munroe Island has been lost, especially in the northwestern regions, and the severity experienced in *Peringalam* and *Cheriyakadavu* Islands with a land depletion of about 12% and 47% respectively. Owing to the environmental issues, the people started to migrate from the Island and as a result the population began to decrease, about 500 in numbers during 2001 and 2011 period and it is against the general population trend of the Kerala state. The lack of livelihood facilities and proper transportation in addition to the ecological challenges forced the residents to leave their homeland and more than 500 households vacated their houses as per the panchayat statistics.

The lithological studies proves that the Munroe Island is formed due to the combined action of marine and fluvial geomorphic processes. The very fertile riverine sediments brought by the Kallada river are accreted at the Ashtamudi backwater confluence at the foothills of *Peringalam* hill. The electrical resistivity profiling and borehole data logs show that there is more than 120 m thick deposition of unconsolidated loose alluvium, which has medium to high compressibility strength. Bathymetric and salinity measurements show that there are many saline pools, which in turn affects both soil stability and groundwater quality. The climate change and its impacts are also contributed for the severity in the existing environmental conditions and affected the socio environmental attributes of the Island.

Though different studies are available for explaining the land degradation issues of the *Mundrothurth*, our study is pointing towards the degradation of the Kallada river by anthropogenic interventions due to sand mining and limits sediment supply, which considerably affected the isostatic conditions and land neutrality. The riverbed pools formed due to the sand mining in the Kallada river has immensely contributed for the present degradation of the *Mundrothuruth*. The bathymetric study and salinity measurements confirmed this observation. The establishment of the ‘Kallada Irrigation Project’ by constructing the Thenmala dam at the lower course of youthful age of the Kallada river caused for the lack of sediment supply through the river discharges. Hence the combined effects of the removal of riverbed sediments and lack of sedimentation contributed for the present land degradation issues in the Munro Island. In this context the human interventions in natural riverine systems by making of dams and reservoirs have also to be considered for addressing the estuarine dynamics and the existence of adjoining lands in other similar places too for proper environmental management. Since the Ashtamudi wetlands and its adjoining area are emerging tourist destinations, sustainable environmental management plans have to be developed as per the existing environmental conditions and socio-economic dimensions.

## Data Availability

The all-data sets used for this study are available with authors and the same may be shared up on reasonable request to the corresponding author.
